# Applying the electronic nose for pre-operative SARS-CoV-2 screening

**DOI:** 10.1007/s00464-020-08169-0

**Published:** 2020-12-02

**Authors:** Anne G. W. E. Wintjens, Kim F. H. Hintzen, Sanne M. E. Engelen, Tim Lubbers, Paul H. M. Savelkoul, Geertjan Wesseling, Job A. M. van der Palen, Nicole D. Bouvy

**Affiliations:** 1 NUTRIM School of Nutrition and Translational Research in Metabolism, Maastricht University, Maastricht, The Netherlands; 2 Department of Surgery, Maastricht University Medical Center, PO Box 5800, 6202 AZ Maastricht, The Netherlands; 3 Department of Medical Microbiology, NUTRIM School of Nutrition and Translational Research in Metabolism, Maastricht University Medical Center, Maastricht, The Netherlands; 4 Department of Respiratory Medicine, Maastricht University Medical Center, Maastricht, The Netherlands; 5 Department of Research Methodology, Measurement, and Data Analysis, University of Twente, Enschede, The Netherlands; 6 Department of Epidemiology, Medisch Spectrum Twente, Enschede, The Netherlands

**Keywords:** COVID-19, Electronic nose, Exhaled air, Volatile organic compounds, Innovative diagnostics

## Abstract

**Background:**

Infection with SARS-CoV-2 causes corona virus disease (COVID-19). The most standard diagnostic method is reverse transcription-polymerase chain reaction (RT-PCR) on a nasopharyngeal and/or an oropharyngeal swab. The high occurrence of false-negative results due to the non-presence of SARS-CoV-2 in the oropharyngeal environment renders this sampling method not ideal. Therefore, a new sampling device is desirable. This proof-of-principle study investigated the possibility to train machine-learning classifiers with an electronic nose (Aeonose) to differentiate between COVID-19-positive and negative persons based on volatile organic compounds (VOCs) analysis.

**Methods:**

Between April and June 2020, participants were invited for breath analysis when a swab for RT-PCR was collected. If the RT-PCR resulted negative, the presence of SARS-CoV-2-specific antibodies was checked to confirm the negative result. All participants breathed through the Aeonose for five minutes. This device contains metal-oxide sensors that change in conductivity upon reaction with VOCs in exhaled breath. These conductivity changes are input data for machine learning and used for pattern recognition. The result is a value between − 1 and + 1, indicating the infection probability.

**Results:**

219 participants were included, 57 of which COVID-19 positive. A sensitivity of 0.86 and a negative predictive value (NPV) of 0.92 were found. Adding clinical variables to machine-learning classifier via multivariate logistic regression analysis, the NPV improved to 0.96.

**Conclusions:**

The Aeonose can distinguish COVID-19 positive from negative participants based on VOC patterns in exhaled breath with a high NPV. The Aeonose might be a promising, non-invasive, and low-cost triage tool for excluding SARS-CoV-2 infection in patients elected for surgery.

In recent months, the world has been overwhelmed by the severe acute respiratory syndrome coronavirus 2 (SARS-CoV-2) outbreak [1–3]. Infection with SARS-CoV-2 causes the Corona Virus Disease (COVID-19) after an incubation period of approximately 5.2 days [4]. The most common clinical manifestations of COVID-19 include fever, cough, fatigue, shortness of breath, and gastrointestinal symptoms [5]. Between 80 and 90% of infected patients are asymptomatic or experience mild symptoms [6, 7], but a small fraction develops more serious complaints such as dyspnea, hypoxemia, and clinical imaging reveals a diffuse involvement of lung parenchyma [7]. Common complications in critically ill COVID-19 patients include acute respiratory distress syndrome, myocardial injury, acute kidney injury, pulmonary embolism, and secondary infection [8, 9]. The case fatality rate appears to be 2.3% [6].

Despite the drastic measures implemented by national governments, the virus has spread quickly around the world. In March 2020, the World Health Organization (WHO) declared the spread of SARS-CoV-2 a pandemic. To date, only remdesivir has been shown to have a significant effect on clinical improvement [10, 11]. Consequently, the WHO’s public advice is to prevent the spread of infection by improving hygiene measures, implementing physical distancing, and applying self-isolation when experiencing symptoms [12]. Effective containment strategies in certain countries have included large-scale testing. Therefore, it is recommended that countries invest in large-scale diagnostic testing for COVID-19 [13].

The current standard and preferred method for diagnosis is a real-time reverse transcription-polymerase chain reaction (RT-PCR) based on a nasopharyngeal and/or oropharyngeal swab. The specificity and sensitivity of this test are very high, but false-positive results sometimes occur due to swab contamination and false-negative results because the non-presence of SARS-CoV-2 in the oropharyngeal environment negatively influences the true sensitivity of the test (66–83%) [7, 14]. Initial false-negative results have been observed and reported based on specific COVID-19 findings on chest CT scans [15, 16]. Therefore, if clinical suspicion is high, a single negative RT-PCR test cannot rule out COVID-19 and the test should be repeated [14]. The relatively high occurrence of false-negative test results makes a new sampling device desirable. Other diagnostic tests include chest CT scans, on which ground-glass opacities appear as typical abnormalities for COVID-19, and analysis of for instance stools or saliva via RT-PCR for detection of current infection [17, 18]. All these tests, however, are expensive and time consuming, require trained personnel, and, in the case of chest CT scans, expose patients to X-rays.

Besides testing of patients with COVID-19-specific clinical manifestations, screening for SARS-CoV-2 also takes place in the elective, pre-operative, asymptomatic population [19]. In general, screening in this population takes place within 48 h prior to the procedure in an outpatient clinic setting using an RT-PCR test. Important reasons for screening are that infected patients have an increased risk for adverse postoperative outcomes, but they might also form a risk for hospital workers, particularly during procedures generating aerosols, and for other hospitalized patients. COVID-19-positive pre-operative patients might be rescheduled, or necessary precautions might be taken to limit the chance of transmission [19].

A promising development in the diagnostic field is based on volatile organic compounds (VOCs). These are gaseous molecules released as a degradation product of metabolic processes in the body whose composition changes directly as a result of pathological processes, such as an infection or a malignancy [20]. Over 850 individual VOCs have already been detected in exhaled breath [21]. Several techniques have been developed to assess these molecules, one of which is the electronic nose (Aeonose). This is a portable, handheld device that can analyze VOC patterns in exhaled breath by their reaction to three metal-oxide sensors incorporated in the device. Since the diagnosis can be made within only sixteen minutes, the test can be considered a point-of-care test. Extensive research with the Aeonose has already been done not only in oncology [22–25] and pre-malignant disorders such as Barrett esophagus [26], but also into infectious diseases such as tuberculosis and in differentiating viral from bacterial respiratory infections in acute COPD exacerbations [27, 28].

In this proof-of-principle study, we investigate the diagnostic performance of the Aeonose in detecting COVID-19 in exhaled breath with nasopharyngeal sampling followed by RT-PCR testing and antibody detection as the reference standard to confirm an earlier SARS-CoV-2 infection.

## Materials and methods

### Study population

This prospective proof-of-principle study was conducted at the Maastricht University Medical Center (MUMC+) from April to June 2020. Participants were recruited via the outpatient clinic for MUMC+ employees with symptoms of COVID-19 or when admitted to the MUMC+ with a confirmed COVID-19 diagnosis. Participants were included if an oropharyngeal or nasopharyngeal swab was collected to perform RT-PCR on. RT-PCR was performed at least 48 h after onset of the first COVID-19 symptoms. If the RT-PCR was negative, blood was collected for the detection of SARS-CoV-2-specific antibodies. This was repeated in most participants after three weeks. Participants were excluded, who were experiencing dyspnea or needed supplemental oxygen.

Participants were divided into two groups: RT-PCR confirmed positive SARS-CoV-2 infection (COVID-19 positive), or RT-PCR confirmed negative SARS-CoV-2 infection without the presence of antibodies (COVID-19 negative). The study protocol was approved by the medical ethical committee of the MUMC+ and was conducted following the Declaration of Helsinki. Oral and written information was given to all eligible participants. Written informed consent was obtained before breath analysis.

### Study design

Measurements were performed at the outpatient clinic for MUMC+ employees with COVID-19 symptoms or at the nursing unit where a SARS-CoV-2 patient was admitted. Inclusion took place directly after performing a specimen collection via a swab or a blood collection for antibodies. Medical students guided the breath collection; they wore personal protective equipment, including coveralls, masks, gloves, and goggles. A short questionnaire was completed to gather information about the onset and type of symptoms. Other recorded information included body mass index, smoking behavior, degree of alcohol consumption, presence of comorbidities, and medication use. Gender, the RT-PCR confirmed diagnosis, and the presence of SARS-CoV-2 specific antibodies were linked to each breathing pattern. Double checking of both RT-PCR and antibody serology was performed by two researchers to avoid misclassification.

### Materials

The electronic, handheld, portable electronic nose (Aeonose) used in this study (Aeonose, The Aeonose Company, Zutphen, the Netherlands) contains three microhotplate metal-oxide sensors: carbon monoxide (AS-MLC), nitrogen dioxide (AS-MLN), and VOC (AS-MLX) sensors. During each measurement, the sensors go through a sinusoidal temperature cycle between 260 and 340 °C; a single breath analysis consists of 32 intervals. Exhaled VOCs react via a redox reaction with these sensors, inducing changes in conductivity and generating numeric patterns. Numeric data are exported to a data center to be stored and analyzed afterward.

Participants breathed for five consecutive minutes through a disposable mouthpiece containing both a carbon filter and a high-efficiency particulate air (HEPA) filter to prevent contamination of the internal tubing. The device contains one lumen with silicon valves to prevent rebreathing of the air in the device (one-way system). A nose clip was placed on the nose of each participant to avoid entry of non-filtered air in the device. Before measuring, the Aeonose was flushed with room air, guided through a carbon filter as well. During each measurement, a video was displayed to distract the participant and to reduce the chance of hyperventilation. Failed breath tests were excluded from analysis; the reason for failure was documented. Four similar Aeonose devices were used for breath analysis. A full-measurement procedure required sixteen minutes. During the first two minutes, the air in the lungs was rinsed to eliminate exogenous VOCs, followed by a three-minute guiding of exhaled air over the sensors. In the remaining eleven minutes, analysis of the data took place, and thereafter, the device was ready for re-use.

### Statistical analysis

Baseline characteristics of the two groups were analyzed with an independent sample *t* test, Fisher’s exact test, and Pearson’s Chi-square test, as appropriate, to identify possible significant differences. A power calculation to obtain an exact power size was not possible for this study, due to the statistical methods used in the Aeonose technology. Previous studies conducted with the Aeonose proposed that at least 25 participants per group are needed to build a disease-specific model. Therefore, we aimed to include at least 25 COVID-19-positive and 25 COVID-19-negative participants in this proof-of-principle study.

During each breath analysis, the following data points were recorded: 64 temperature values × 36 measurement cycles × 3 sensors. To minimize and eliminate inter-device differences, data were pre-processed including standardization. Pre-processed data were compressed using a Tucker3-like solution, resulting in a single vector of limited size per participant. These vectors were, together with the participant’s diagnosis, used to train an artificial neural network (ANN). This ANN is a computational system based on multiple layers of associations, comparable to the neural network of the human brain, and therefore, capable of teaching itself. By using the data analysis package Aethena (The Aeonose Company), combinations of several pre-processing techniques, vector lengths, and network topologies were investigated to optimize results. Classifier techniques like random forest and logistic regression were applied as well. “Leave-10%-out” cross-validation was applied to prevent the fitting of the data on artifacts instead of breath profile classifiers. All data were categorized when processed by Aethena. The individual binary predictive values were presented in a scatter plot and a receiver operating characteristic curve (ROC-curve). 95% Confidence intervals are presented. More details on data analysis via the Aeonose have already been published [29]. Subsequently, we added clinical and demographic variables that differed between COVID-19-positive and COVID-19-negative participants, together with the value obtained from the Aeonose in a multivariate logistic regression model, using a forward stepwise (conditional) approach, to improve the predictive value of having COVID-19.

## Results

Breath samples were obtained from 219 participants, 57 of which were COVID-19 positive and 162 COVID-19 negative. In three percent, the breath test had failed due to dyspnea or technical difficulties. No adverse events were observed during breath analysis. The main characteristics of all participants are summarized in Table 1. There were significantly more males in the COVID-19-negative group (*p* = 0.001). The antibiotic use in the past three months was also higher in this group (*p* = 0.023). In the COVID-19-positive group, the incidence of prior or current malignancy was significantly higher (*p* = 0.017).

**Table 1:** Baseline characteristics of the total study cohort (*n* = 219)

Parameter	COVID-19 positive (*n* = 57)	COVID-19 negative (*n* = 162)	*p* value
Male gender, *n* (%)	35 (61.4)	135 (83.3)	0.001
Age (years), mean ± SD	39.44 ± 13.9	41.21 ± 12.9	0.384
BMI (kg/m^2^), mean ± SD	25.9 ± 3.8	25.6 ± 5.2	0.663
Smoking status			
Never, *n* (%)	40 (70.2)	118 (72.8)	0.732
Former/current, *n* (%)	17 (29.8)	44 (27.2)	-
Alcohol (U/week), mean ± SD	1.4 ± 2.1	2.1 ± 2.6	0.062
Comorbidities			
Hypertension, *n* (%)	6 (10.5)	15 (9.3)	0.796
Diabetes mellitus, *n* (%)	2 (3.5)	4 (2.5)	0.652
Coronary disease, *n* (%)	0	2 (1.2)	1.00
COPD/asthma, *n* (%)	2 (3.5)	10 (6.2)	0.736
Malignancy, *n* (%)	4 (7.0)	1 (0.6)	0.017
Kidney disorders, *n* (%)	1 (1.8)	0	0.260
Medication use			
PPI, *n* (%)	1 (1.8)	9 (5.6)	0.460
NSAID, *n* (%)	1 (1.8)	15 (9.3)	0.076
Corticosteroid, *n* (%)	2 (3.5)	5 (3.1)	1.00
ACE inhibitor, *n* (%)	3 (5.3)	1 (0.6)	0.055
Angiotensin receptor blocker, *n* (%)	1 (1.8)	2 (1.2)	1.00
Antibiotics in the past 3 months, *n* (%)	0	13 (8.0)	0.023

On the day of inclusion, participants experienced COVID-19 complaints about an average of 13.4 (± 12.4) days in the COVID-19-positive group and 12 (± 15.9) days in the COVID-19-negative group. Mean cycle threshold (Ct) value in the COVID-19 positive group was 31 (range 18–40). The incidence of the specific symptoms is displayed in Table 2.

**Table 2:** The incidence of COVID-19-specific symptoms in the total study cohort

Symptom	COVID-19 positive (*n* = 57)	COVID-19 negative (*n* = 162)	*p* value
Coughing, *n* (%)	24 (42.1)	85 (52.5)	0.218
Dyspnea, *n* (%)	16 (28.1)	34 (21.0)	0.277
Fever, *n* (%)	27 (47.4)	33 (20.4)	< 0.001
Sore throat, *n* (%)	23 (40.4)	94 (58.0)	0.030
Increased sputum production, *n* (%)	10 (17.5)	45 (27.8)	0.156
Fatigue, *n* (%)	39 (68.4)	74 (45.7)	0.003
Myalgia, *n* (%)	25 (43.9)	77 (47.5)	0.004
Headache, *n* (%)	32 (56.1)	44 (47.5)	0.284
Diarrhea, *n* (%)	10 (17.5)	23 (14.2)	0.526
Nausea/vomiting, *n* (%)	4 (7.0)	19 (11.7)	0.452
Anosmia/ageusia, *n* (%)	5 (8.8)	4 (2.5)	0.054

**Fig. 1:**
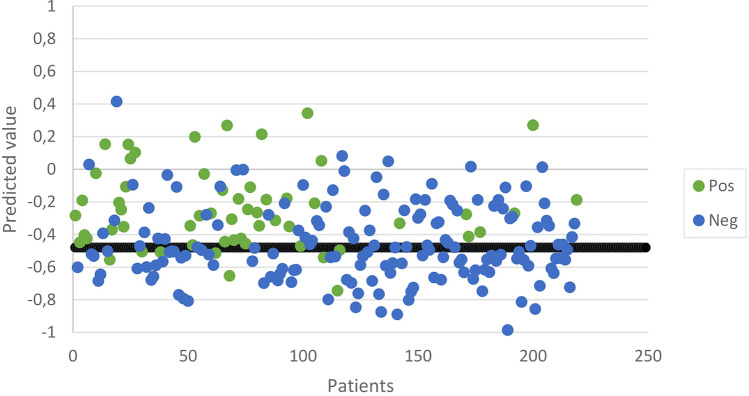
Scatterplot of individual predictive values of each participant. Values > − 0.48 are scored as COVID-19 positive. In green, COVID-19-positive participants are represented and in blue COVID-19-negative participants (Color figure online)

**Fig. 2:**
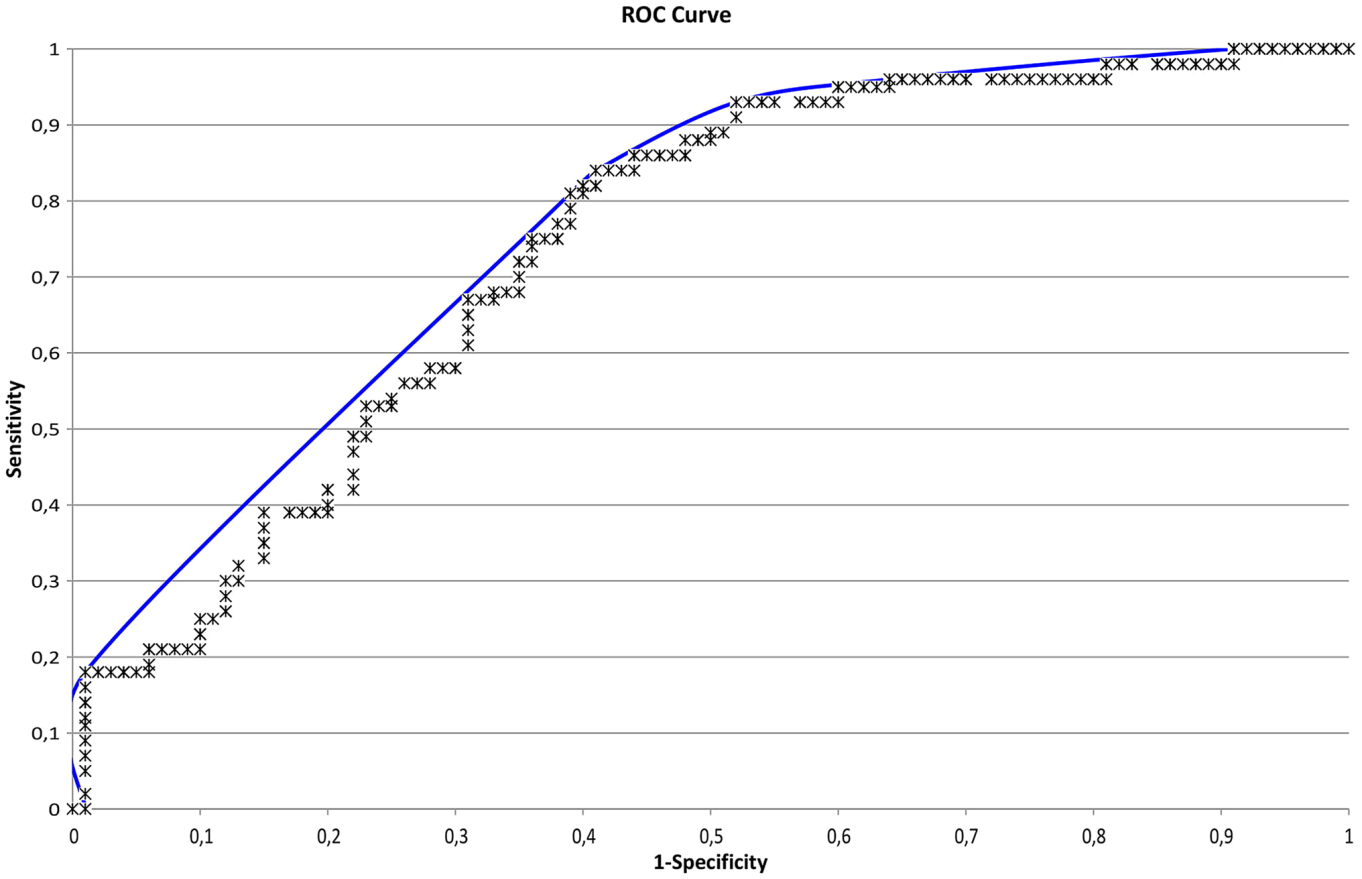
Receiver operating characteristic (ROC) curve illustrates the diagnostic performance of the Aeonose. The area under the curve is 0.74

The prevalence of COVID-19 in our study population was 26%. The composition of the exhaled breath differed significantly between COVID-19-positive and negative participants with an area under the curve (AUC) of 0.74. To obtain an acceptable balance between various diagnostic parameters in relation to clinical desirability, the threshold was set to -0.48. This resulted in a sensitivity of 0.86 (95% CI 0.74–0.93) with a negative predictive value (NPV) of 0.92 (95% CI 0.84–0.96). The specificity and positive predictive value (PPV) were 0.54 (95% CI 0.46–0.62) and 0.40 (95% CI 0.31–0.49), respectively. The overall accuracy was 0.62. In Figs. 1 and 2, the scatterplot and the receiver operating characteristic (ROC) curve are illustrated. This prediction model was cross validated (tenfold).

To obtain a higher sensitivity, the threshold was set to 0.16 (i.e., probability of 16% or higher to be COVID-19 positive, based on a univariate logistic regression model). This resulted in a sensitivity of 0.95 (95% CI 0.86–0.99), with a NPV of 0.95 (95% CI 0.88–0.99). The specificity and PPV were, respectively, 0.39 (95% CI 0.32–0.47) and 0.35 (95% CI 0.28–0.43).

Five clinical variables from Tables 1 and 2 were added to multivariate logistic regression analysis, resulting in the model as displayed in Table 3. When applying the multivariate logistic regression model together with the value from the machine-learning classifier (− 1 to + 1) of the exhaled breath in the same study population, the AUC improved to 0.84. Corresponding sensitivity and NPV were 0.93 (95% CI 0.84–0.98) and 0.96 (95% CI 0.90–0.98), respectively, with a specificity of 0.56 (95% CI 0.48–0.64) and a PPV of 0.43 (95% CI 0.34–0.52). The threshold was set to 0.14.

**Table 3:** The results of the multivariate logistic regression analysis for diagnosing COVID-19

Variable	Odds ratio	*B*	*p* value
Female sex	3.4 (1.5–7.6)	1.2	0.003
Fever	4.0 (1.9–8.6)	1.4	0.000
Sore throat	0.39 (0.18–0.81)	− 0.95	0.012
Fatigue	2.5 (1.1–5.5)	0.92	0.021
Anosmia/ageusia	5.0 (0.97–25.7)	1.6	0.055
Aeonose classification value	49.4 (9.7–252.7)	3.9	0.000

Data are presented as odds ratio (95% confidence interval). Constant is − 0.603

*B* regression coefficient

## Discussion

In this proof-of-principle study, we investigated the diagnostic performance of the Aeonose in COVID-19 detection in exhaled breath. RT-PCR results together with the presence of SARS-CoV-2-specific antibodies in the blood were used as a reference standard. Our study outcome suggests that breath analysis by the Aeonose has the potential to become a quick, low-cost, and non-invasive triage test for COVID-19. With an NPV of 92%, the Aeonose was able to differentiate between COVID-19-positive and negative VOC patterns. When adding clinical and demographic relevant variables to the machine-learning classifier via multivariate logistic regression analysis, the NPV improved to 96%.

The use of VOC analysis through an electronic nose has been described extensively. A recent study of Peters et al. showed that Aeonose technology can detect a Barrett esophagus in patients with or without gastroesophageal reflux disease with a sensitivity of 91% and a specificity of 74%. In the future, the Aeonose might play an important role in the primary care setting as non-invasive screening for Barrett esophagus [26]. In the detection of tuberculosis, comparable Aeonose technology has shown its good diagnostic accuracy as well: a sensitivity of 93.5% and a specificity of 85.3% were found in distinguishing tuberculosis patients from healthy controls in Bangladesh [30].

Our study has several important strengths. This is the first study to illustrate the diagnostic performance of an electronic nose in detecting COVID-19. Compared to currently used diagnostic tools, the Aeonose is much cheaper, results are obtained faster because of real-time analysis, and no specific personnel is needed since the device is easy to use. Another strength is the relatively low dropout rate of approximately 3%, which is in line with findings from other Aeonose studies in patients with respiratory symptoms where a 0% dropout was found [28]. Most of our study participants were MUMC+ employees, experiencing relatively mild COVID-19 symptoms without dyspnea or in need of supplemental oxygen since these were exclusion criteria. The low dropout rate shows that in this study population, the Aeonose is a well tolerable and suitable diagnostic tool. In addition, breath sampling was supported by dedicated medical students which can explain the low dropout rate.

However, this study also has some limitations. The most important limitation is the necessity of the RT-PCR test as a reference standard (because the accuracy of the RT-PCR is limited by the non-presence of SARS-CoV-2 in the nasopharyngeal cavity) in training the algorithm since this is the current standard to diagnose COVID-19. The sensitivity of the whole RT-PCR procedure (including sampling) is relatively low (66–83%), resulting in the risk of missing infected participants and thereby developing an inaccurate algorithm. We aimed at mitigating this problem by double checking all COVID-19-negative participants; a COVID-19-negative participant was only included in the algorithm if RT-PCR was negative in combination with the absence of SARS-CoV-2-specific antibodies in the blood. Most false-negative RT-PCR results can be explained in two ways: the swab sampling was too early or too late in the disease stage, or the sampling in the oropharynx and/or nasopharynx was not deep enough. In both examples, the viral load might be too low resulting in a false-negative RT-PCR result [31]. Since breath analysis does not depend on deep sampling, we assume that the Aeonose is capable of overcoming at least one of both examples. Even minor changes in VOC composition as a result of COVID-19 might be detected by the Aeonose. Another limitation might be the timing of antibody detection. Although the seroconversion rate for SARS-CoV-2 antibodies is high (93.1%), the median day for seroconversion for both IgM and IgG is 13 days after onset of symptoms [32, 33]. In most of our study participants, the presence or absence of antibodies was determined twice with an interval of three weeks. However, in some participants, the antibody detection was not repeated within three weeks after the absence of antibodies risking false-negative outcomes.

Another limitation is that alcohol in the vicinity of the device disturbs the sensors resulting in non-interpretable data. Using sodium hypochlorite 0.6% as a disinfectant can prevent this. A final limitation is that this study has not been carried out during the influenza season. The ability of the Aeonose to discriminate between various common respiratory viruses is not clear.

The high NPV of this study implicates that the Aeonose can play an important role as a triage diagnostic tool for excluding a SARS-CoV-2 infection. A large prospective validation study is planned to further train and finally validate the algorithm in recognizing COVID-19 in symptomatic participants. With this validated algorithm, the diagnostic accuracy of excluding COVID-19 can be investigated in an asymptomatic population to test the ability of the Aeonose as a screening tool in the general population and in the elective pre-operative population.

In conclusion, this proof-of-principle study demonstrates that the Aeonose has the capacity to distinguish COVID-19-positive from COVID-19-negative participants based on specific VOC patterns in exhaled breath with a high NPV of 0.92, which can further increase to 0.96 when adding clinical relevant variables to the machine-learning classifier via multivariate logistic regression analysis. Rapid availability of the diagnosis, combined with the low costs and a non-invasive aspect of this device suggests that breath analysis via the Aeonose might be a promising triage tool for excluding COVID-19 in the near future.

## References

[1] Coronaviridae Study Group of the International (2020). The species severe acute respiratory syndrome-related coronavirus: classifying 2019-nCoV and naming it SARS-CoV-2. Nat Microbiol.

[2] Zhu N, Zhang D, Wang W, Li X, Yang B, Song J (2020). A novel coronavirus from patients with pneumonia in China, 2019. N Engl J Med.

[3] Wu F, Zhao S, Yu B, Chen YM, Wang W, Song ZG (2020). A new coronavirus associated with human respiratory disease in China. Nature.

[4] Rothan HA, Byrareddy SN (2020). The epidemiology and pathogenesis of coronavirus disease (COVID-19) outbreak. J Autoimmun.

[5] Zhang JJ, Dong X, Cao YY, Yuan YD, Yang YB, Yan YQ (2020). Clinical characteristics of 140 patients infected with SARS-CoV-2 in Wuhan, China. Allergy.

[6] Novel Coronavirus Pneumonia Emergency Response Epidemiology (2020). The epidemiological characteristics of an outbreak of 2019 novel coronavirus diseases (COVID-19) in China. Zhonghua Liu Xing Bing Xue Za Zhi..

[7] Pascarella G, Strumia A, Piliego C, Bruno F, Del Buono R, Costa F (2020). COVID-19 diagnosis and management: a comprehensive review. J Intern Med.

[8] Siordia JA (2020). Epidemiology and clinical features of COVID-19: a review of current literature. J Clin Virol.

[9] Huang C, Wang Y, Li X, Ren L, Zhao J, Hu Y (2020). Clinical features of patients infected with 2019 novel coronavirus in Wuhan, China. Lancet.

[10] Jin YH, Cai L, Cheng ZS, Cheng H, Deng T, Fan YP (2020). A rapid advice guideline for the diagnosis and treatment of 2019 novel coronavirus (2019-nCoV) infected pneumonia (standard version). Mil Med Res.

[11] Wise J (2020). Covid-19: Remdesivir is recommended for authorisation by European Medicines Agency. BMJ.

[12] World Health Organization (2020). Coronavirus disease (COVID-19) advice for the public 2020.

[13] Cheng MP, Papenburg J, Desjardins M, Kanjilal S, Quach C, Libman M (2020). Diagnostic testing for severe acute respiratory syndrome-related coronavirus-2: a narrative review. Ann Intern Med.

[14] Long C, Xu H, Shen Q, Zhang X, Fan B, Wang C (2020). Diagnosis of the coronavirus disease (COVID-19): rRT-PCR or CT?. Eur J Radiol.

[15] Xie X, Zhong Z, Zhao W, Zheng C, Wang F, Liu J (2020). Chest CT for typical 2019-nCoV pneumonia: relationship to negative RT-PCR testing. Radiology.

[16] Ai T, Yang Z, Hou H, Zhan C, Chen C, Lv W (2020). Correlation of chest CT and RT-PCR testing in coronavirus disease 2019 (COVID-19) in China: a report of 1014 cases. Radiology.

[17] Iyer M, Jayaramayya K, Subramaniam MD, Lee SB, Dayem AA, Cho SG (2020). COVID-19: an update on diagnostic and therapeutic approaches. BMB Rep.

[18] Xie J, Ding C, Li J, Wang Y, Guo H, Lu Z (2020). Characteristics of patients with coronavirus disease (COVID-19) confirmed using an IgM-IgG antibody test. J Med Virol.

[19] Hojaij FC, Chinelatto LA, Boog GHP, Kasmirski JA, Lopes JVZ, Sacramento FM (2020). Surgical practice in the current COVID-19 pandemic: a rapid systematic review. Clinics.

[20] Haick H, Broza YY, Mochalski P, Ruzsanyi V, Amann A (2014). Assessment, origin, and implementation of breath volatile cancer markers. Chem Soc Rev.

[21] de Lacy CB, Amann A, Al-Kateb H, Flynn C, Filipiak W, Khalid T (2014). A review of the volatiles from the healthy human body. J Breath Res.

[22] Schuermans VNE, Li Z, Jongen A, Wu Z, Shi J, Ji J (2018). Pilot study: detection of gastric cancer from exhaled air analyzed with an electronic nose in Chinese patients. Surg Innov.

[23] van de Goor R, van Hooren M, Dingemans AM, Kremer B, Kross K (2018). Training and validating a portable electronic nose for lung cancer screening. J Thorac Oncol.

[24] Waltman CG, Marcelissen TAT, van Roermund JGH (2018). Exhaled-breath testing for prostate cancer based on volatile organic compound profiling using an electronic nose device (aeonose): a preliminary report. Eur Urol Focus.

[25] van Keulen KE, Jansen ME, Schrauwen RWM, Kolkman JJ, Siersema PD (2020). Volatile organic compounds in breath can serve as a non-invasive diagnostic biomarker for the detection of advanced adenomas and colorectal cancer. Aliment Pharmacol Ther.

[26] Peters Y, Schrauwen RWM, Tan AC, Bogers SK, de Jong B, Siersema PD (2020). Detection of Barrett's oesophagus through exhaled breath using an electronic nose device. Gut.

[27] Saktiawati AMI, Stienstra Y, Subronto YW, Rintiswati N, Gerritsen JW (2019). Sensitivity and specificity of an electronic nose in diagnosing pulmonary tuberculosis among patients with suspected tuberculosis. PLoS ONE.

[28] van Geffen WH, Bruins M, Kerstjens HA (2016). Diagnosing viral and bacterial respiratory infections in acute COPD exacerbations by an electronic nose: a pilot study. J Breath Res.

[29] Kort S, Brusse-Keizer M, Gerritsen JW, van der Palen J (2017). Data analysis of electronic nose technology in lung cancer: generating prediction models by means of Aethena. J Breath Res.

[30] Bruins M, Rahim Z, Bos A, van de Sande WW, Endtz HP, van Belkum A (2013). Diagnosis of active tuberculosis by e-nose analysis of exhaled air. Tuberculosis.

[31] Wikramaratna P, Paton R, Ghafari M, Lourençp J (2020). Estimating false-negative detection rate of SARS-CoV-2 by RT-PCR. arXiv.

[32] Zhao J, Yuan Q, Wang H, Liu W, Liao X, Su Y (2020). Antibody responses to SARS-CoV-2 in patients of novel coronavirus disease 2019. Clin Infect Dis.

[33] Long Q-X, Deng H-J, Chen J, Hu J, Liu B-Z, Liao P (2020). Antibody responses to SARS-CoV-2 in COVID-19 patients: the perspective application of serological tests in clinical practice. medRxiv.

